# Suicidal Behavior and Alcohol Abuse

**DOI:** 10.3390/ijerph7041392

**Published:** 2010-03-29

**Authors:** Maurizio Pompili, Gianluca Serafini, Marco Innamorati, Giovanni Dominici, Stefano Ferracuti, Giorgio D. Kotzalidis, Giulia Serra, Paolo Girardi, Luigi Janiri, Roberto Tatarelli, Leo Sher, David Lester

**Affiliations:** 1 Department of Neuroscience, Mental Health and Sensory Functions, Suicide Prevention Center, Sant’Andrea Hospital, Sapienza University of Rome, Rome 00189, Italy; E-Mails: gianluca.serafini@uniroma1.it (G.S.); innamorati.marco@libero.it (M.I.); gjdominus@hotmail.it (G.D.); stefano.ferracuti@uniroma1.it (S.F.); giorgio.kotzalidis@uniroma1.it (G.D.K.); giuliaserra@gmail.com (G.S.); paolo.girardi@uniroma1.it (P.G.); roberto.tatarelli@uniroma1.it (R.T.); 2 McLean Hospital, Harvard Medical School, Belmont, MA 02478, USA; 3 Department of Psychiatry, Catholic University Medical School, Largo F. Vito 1, Rome 00168, Italy; E-Mail: luigi_janiri@fastwebnet.it; 4 Department of Psychiatry, Columbia University, New York, NY 10032, USA; E-Mail: drleosher@gmail.com; 5 The Richard Stockton College of New Jersey, Pomona, NJ 08240-0195, USA; E-Mail: david.lester@richardstockton.edu

**Keywords:** alcohol abuse, suicidal behavior, psychiatric disorders, pathophysiology, prevention

## Abstract

Suicide is an escalating public health problem, and alcohol use has consistently been implicated in the precipitation of suicidal behavior. Alcohol abuse may lead to suicidality through disinhibition, impulsiveness and impaired judgment, but it may also be used as a means to ease the distress associated with committing an act of suicide. We reviewed evidence of the relationship between alcohol use and suicide through a search of MedLine and PsychInfo electronic databases. Multiple genetically-related intermediate phenotypes might influence the relationship between alcohol and suicide. Psychiatric disorders, including psychosis, mood disorders and anxiety disorders, as well as susceptibility to stress, might increase the risk of suicidal behavior, but may also have reciprocal influences with alcohol drinking patterns. Increased suicide risk may be heralded by social withdrawal, breakdown of social bonds, and social marginalization, which are common outcomes of untreated alcohol abuse and dependence. People with alcohol dependence or depression should be screened for other psychiatric symptoms and for suicidality. Programs for suicide prevention must take into account drinking habits and should reinforce healthy behavioral patterns.

## 1. Introduction

The estimated global burden of suicide is a million deaths every year [1], and a policy statement produced by WHO in response to this [2] has urged countries to implement suicide prevention policies. The estimated annual mortality from suicide is 14.5 suicides per 100,000 people, about one death every 40 seconds [1]. Self-inflicted deaths were the tenth leading cause of death worldwide and accounted for 1.5% of all deaths [3]. Suicide rates differ by sex, age, ethnic origin and death registration system, as well as by region and over time.

Globally, alcohol consumption has increased in recent decades, with all or most of that increase occurring in developing countries. Alcohol consumption has health and social consequences *via* intoxication (drunkenness), dependence (habitual, compulsive and long-term drinking), and biochemical effects. In addition to chronic diseases that may affect drinkers after many years of heavy use, alcohol contributes to traumatic outcomes that kill or disable at a relatively young age, resulting in the loss of many years of life to death or disability. There is increasing evidence that, aside from the volume of alcohol consumed, the pattern of the drinking is relevant for health outcomes. Overall, there is a causal relationship between alcohol consumption and more than 60 types of diseases and injuries. Alcohol is estimated to cause about 20–30% of cases of oesophageal cancer, liver cancer, cirrhosis of the liver, homicide, epilepsy and motor vehicle accidents. Alcohol had been used by most people in the Americas, Europe, Japan, and New Zealand, with smaller proportions in the Middle East, Africa, and China [4].

In the last 45 years, suicide rates have increased by about 60% worldwide, with global suicide figures potentially reaching 1.5 million deaths by the year 2020 [5]. Although traditionally suicide rates have been highest among elderly males, rates among young people have been increasing to such an extent that they are now the group at highest risk in roughly one-third of nations, in both developed and developing countries. Mental disorders (particularly depression and substance abuse) are often associated with cases of suicide. However, suicide results from many complex socio-cultural factors and is likely to occur particularly during periods of socioeconomic, family and personal crisis situations (e.g., loss of a loved one, employment, dignity, *etc.*).

The existence of a link between alcohol use and suicide was known to Kraepelin [6]. This link has been advanced more convincingly since the mid-1960s [7–9] and confirmed in recent years [10–13]. Most research on alcohol use and suicide has focused on suicidal ideation or attempted suicide [14] rather than completed suicide, because of the methodological difficulties involved in investigating completed suicide. However, it is important to realize that, despite some overlap, suicide attempters and completers show demographic, personality, and clinical differences [11].

Suicide is held to be a complication of psychiatric disorder [10,15]. Mood [10,16,17], anxiety [18] and schizophrenia-spectrum disorders [16,19,20] have been found to constitute independent risk factors for suicidal behavior. Additionally, co-morbid psychiatric disorders are found to be common in patients with alcohol use disorders [21–24]. Alcohol use is highly prevalent worldwide, and suicide is highly prevalent in populations of patients with alcohol use disorders. However, co-morbid psychopathology is neither sufficient nor necessary for this association [14]. Alcohol use and suicide are intimately linked, but they are both complex phenomena, springing from a multitude of factors. Menninger conceptualized addiction itself both as a form of *chronic suicide* and as a factor involved in *focal suicide* (deliberate self-harming accidents) [25].

The focus of this paper is to provide a broad overview of the much debated relationship between alcohol and suicide. This study starts with the assumption that, according to research, the suicide rate is substantially elevated among alcoholics and that suicide is a cause of death for a substantial percentage of alcoholics. This review is based on the assumption also that alcohol use, particularly heavy use and alcohol dependence, is highly associated with suicide in three ways: (1) alcohol, through its disinhibiting effects, is related to suicide attempts and completions; (2) individuals with alcohol use disorders are at an increased risk of suicide as compared to the population at large; and (3) at the population level (nationally and internationally), alcohol consumption is correlated with the suicide rate [26]. This review updates and critically considers data on the alcohol use-suicidal behavior link and suggests implications for future research.

## 2. Methods

Printed documentation was searched in the MedLine and PsychInfo electronic databases. The following limits were set for the MEDLINE search: adults 19+ years, publication date from 1991 to 2009, articles with abstracts, and articles written in English. A variety of strategies were used to conduct a more detailed search. The most efficient search strategy was: (suicide OR suicide attempt OR ideation) AND (epidemiology OR rates OR trends OR incidence) AND (alcohol use OR alcohol intoxication OR alcohol drinking OR alcoholism OR alcohol use disorder OR alcohol dependent) AND (“prevention” OR “intervention” OR “implications for the future”).

To ensure that relevant publications were not missed, using the same limits specified earlier, each of the following terms was searched individually with “AND suicide”: alcohol use, alcohol use disorder, alcohol intoxication, alcohol drinking, alcoholism, alcohol acute dependent, inebriation, ethanol and alcohol consumption. PsycINFO was also searched using the same limits mentioned above. Duplicate articles were removed. Abstracts that did not explicitly mention suicide and alcohol use and articles that did not provide levels/measures of alcohol use specifically for suicides were excluded.

## 3. The Alcohol-Suicide Link: A Bond Difficult to Disentangle

Alcohol abuse is the commonest type of substance dependence worldwide. Suicide is major public health issue. Therefore, given the enormous socioeconomic burden of the latter, investigating their possible relationships is mandatory. However, it is difficult to establish the right questions to ask. Does alcohol induce suicide? Does alcohol prevent suicide in some cases? Does alcohol-related misconduct increase the risk of suicide? Do people drink to ensure the courage needed to engage in their suicidal act? Is alcohol part of the method for the suicidal act? Edgar Allen Poe’s drinking to death is illustrative of the latter. How should we define suicide? Is a traffic accident secondary to dangerous driving after drinking alcohol an accident or a suicide attempt? How many of the fatalities, occurring after such events, are to be attributed to suicidal intent? We will here clarify some terms regarding alcohol use and suicide to help understand their relationship.

### 3.1. Alcohol and Suicide: Definitional Issues

In the Diagnostic and Statistical Manual of Mental Disorders, Fourth Edition-Text Revision (DSM-IV-TR) [27], alcohol is one of eleven classes of substances for which a substance-related disorder may be considered when effects secondary to its use arise. Alcohol-related disorders are classified as alcohol use disorder, alcohol-induced disorders, and alcohol-related disorder not otherwise specified. The latter is diagnosed when the criteria for the former two are not fulfilled. Alcohol use disorder comprises alcohol dependence and alcohol abuse, which may be diagnosed only in the absence of dependence. Alcohol dependence may be defined by any three or more of the following in a 12-month period: tolerance, defined by the need to increase the dose in order to maintain its effect and/or the decrease of the effect with continued use at the same dose; withdrawal, defined as the emergence of an alcohol-characteristic syndrome upon suspension or steep dose reduction, that can be reversed by alcohol or soothed by similarly acting substances (for example, benzodiazepines); intake at higher doses or for more prolonged periods than those intended by the subject; failed attempts or no desire to reduce or control substance use; considerable time wasted in seeking alcohol or recovering from its effects; impaired social, professional and recreational activities because of the use of alcohol; and continued use despite awareness of the problems related to the use of alcohol. Abuse is defined as recurrent use of alcohol resulting in impairment in interpersonal, social and professional functioning, in the face of physical danger or legal problems, and continued despite all the above risks. Craving refers to compulsive drug-seeking.

Alcohol-induced disorders comprise delusions and delirium, memory disorder and sleep disorders appearing during intoxication or withdrawal and, in addition, anxiety, mood and psychotic disorders, dementia, and sexual dysfunction related to both acute and chronic alcohol use. These disorders also include the typical microzooptic hallucinations, delirium tremens and Korsakoff’s syndrome, which may occur in the alcohol withdrawal syndrome.

Suicide is an act performed by someone who intends to kill him/herself. Acts having a high probability of death, whether performed consciously or not, without any self-killing intent, are considered to be accidents even though many of these acts may result in death. A suicide attempt is an effort to kill oneself that does not result in death. Attempts are subdivided by their grade of lethality. The most serious are those associated with high lethality. Although the lethality of a suicidal act is related to the suicidal intent of the individual, there is not a perfect correspondence [28]. Self-harm is an act that is not intended to kill the person, but rather to deliberately cause damage, such as cutting or self-mutilation. Sometimes, permanent effects result, and accidental death is always a possibility.

This fuzzy picture led suicidologists to try to fill the gap of a lack of an official nomenclature for suicide and related behavior. In 1996, O’Carroll *et al.* [29] proposed a classification based on three characteristics, that is, intent to die, evidence of self-inflicted injury and outcome (injury, no injury and death). Their approach was followed in subsequent revisions of suicide terminology. The 1998 WHO document on the prevention of neurological, mental and psychosocial disorders [30] confirmed the 1986 Working Group definition with some rewording and, subsequently, De Leo *et al.* [28], after a sound critique of past definitions, proposed a slightly, but significantly modified, redefinition of the WHO 1986 classification, which is in line with that of O’Carroll *et al.* [29], that is, “Suicide is an act with fatal outcome, which the deceased, knowing or expecting a fatal outcome, has initiated and carried out with the purpose of bringing about wanted changes”.

Silverman *et al.* [31] revised O’Carroll’s nomenclature, focusing on suicide-related ideation, communication and behavior. They based their definitions on the presence or absence of suicidal intent and the presence or absence of injury. They purposely avoided adding a third domain of lethality (or degree of injury) because currently there is a lack of consensus for defining lethality. According to this classification, suicide is a fourth order event in a set where the first order (*i.e.*, the name of the set) is represented by Self-Injurious Thoughts and Behaviors. Subsets of the set are risk-taking thoughts and behaviors and suicide-related thoughts and behaviors. The former is subdivided into immediate or remote risk for life and further subclassified, as all other subsets of the classification, according to the outcome, that is, non injury, injury (no matter how severe) and death. The latter subset is further subclassified into suicide-related ideation, suicide-related communications, and suicide-related behaviors. Ideation is further subclassified according to intent, that is, absent, undetermined or present (independent of its degree), and may be casual, transient, passive, active, or persistent. The second subset is suicide-related communications, again subdivided into with, without or undetermined intent, and each of these is further subdivided into fourth-order subsets namely, verbal or nonverbal; passive or covert lumped together to constitute suicide threat, and a proposed method of achieving a potentially self-injurious outcome, constituting a suicide plan. The third second-order set is suicide-related behaviors, with third-order subsets arranged according to intent, which is none, undetermined or some (Silverman *et al.* [31] did not add “or more”, but this is implicit), and each third order subset is composed of three fourth-order sets according to outcome, with no injury, injury or death.

### 3.2. Alcohol and Suicide: Epidemiological Observations

Recent findings from the National Epidemiological Survey on Alcohol and Related Conditions (NESARC) [32] indicate that the 12-month prevalence of DSM-IV-TR alcohol dependence in the adult population in USA is 3.8% and that of alcohol abuse 4.7% [33]. This means that, every year, 8.5% of the adult US population in USA has an alcohol use disorder [33].

A meta-analysis of cohort studies [34] showed that both alcohol and drug use disorders are strongly associated with suicide. Heavy alcohol consumers had a five-fold higher risk of suicide than social drinkers. The National Comorbidity Survey (NCS), focusing on adolescents, found no causal relationships between alcohol drinking behavior in general and suicide attempts, but reported a significant association between alcohol-related disorders and suicide attempts [35]. An increase in suicidal thinking, but not attempts, with alcohol abuse was found in the NCS-R population, but the strength of the association between suicide and asthma condition in the subpopulation with asthma was weakened by alcohol abuse [36]. An Italian study, carried-out in the context of the European Study of the Epidemiology of Mental Health Disorders, a part of the WHO World Mental Health Survey Initiative, showed that suicidal thinking and planning are increased by having an alcohol-related disorder, but that this increase was not paralleled by an increase in suicide attempts [37]. Psychological autopsy studies have confirmed the association between alcohol use and suicide [38], despite lumping alcohol with other drugs due to sample size limitations [39]. In spite of their limited statistical power, psychological autopsy studies in different nations over different decades have consistently documented that mood and substance use disorders, particularly alcoholism, are the most prevalent disorders in suicides [40–43]. Methodologically different psychological autopsy follow-up and case-control studies [34,44,45] indicate that alcohol- and other drug-related disorders are risk factors for suicide [39,46–49].

The association between alcohol use and suicide has also been documented using aggregate studies of alcohol consumption in various countries [50,51]. This association varies with age [52], gender [53] and country [51]. Results from time-series analyses on aggregate level data from several European countries indicates a stronger effect of alcohol consumption on suicide in low consumption countries than in high consumption countries [54–58].

Murphy [59] speculated that the gender-related differences he found in his previous studies [60,61] were due to societal attitudes towards women and to different thinking in women that brought them to seek help and decrease their social isolation. In fact, what was rendering men vulnerable to the effect of alcohol on suicide (independence and loss of interpersonal support) was opposite to what women endorsed (interrelatedness and help seeking). Obviously, Murphy’s finding is limited to Western societies as trends may be reversed in non-Western societies, such as Papua New Guinea [62]. However, in a later study conducted in Canterbury, New Zealand, Conner *et al.* [63] failed to detect an effect of gender in mediating the association between alcohol dependence and serious suicide attempts. The issue is still open.

The lifetime prevalence suicide of attempts in patients with alcohol dependence is high. About 40% of all patients seeking treatment for alcohol dependence report at least one suicide attempt at some point in their lives [64–66]. Impulsive suicide attempts are common in patients with an alcohol use disorder [67,68]. However, whether a history of suicide attempts is related to the risk for relapse in alcohol-dependent patients is still a matter of debate.

In a study of 450 alcohol-dependent men conducted in the mid-eighties, suicide attempts predicted increased alcohol-related problems at one-year follow-up [69], but this has not been confirmed in later studies [70–72].

### 3.3. Alcohol and Suicide: the Search for Causal Relationships. The Size of the Association, the Role of Psychiatric Disorders and Impulsiveness

Various classical studies found an excess of suicide among alcoholics [73–80]. Beck and Steer [81] and Beck *et al.* [82] found that alcoholism was the strongest single predictor of subsequent completed suicide in a sample of attempted suicides.

In 1997, Harris and Barraclough, in their unusually comprehensive meta-analysis analyzed 32 papers related to alcohol dependence and abuse, comprising a population of over 45,000 individuals [34]. They found that combining the studies gave a suicide risk almost six times that expected but with variation of 1–60 times. Specifically, they found that the suicide risk for females was very much greater than for males, about 20 times that expected compared with four for males. Suicide risk among alcohol-dependent individuals has been estimated to be 7% (comparable with 6% for mood disorders; [83]). Of 40,000 Norwegian conscripts followed prospectively over 40 years, the probability of suicide was 4.76% (relative risk +6.9) among those classified as alcohol abusers compared with 0.63 for non-drinkers [84]. Similar finding have been made worldwide [85]. Murphy *et al.* studied 50 suicides and found that an alcohol use disorder was the primary diagnosis in 23% and a co-occurring diagnosis in 37% [86]. Conwell *et al.* performed a study in New York City and reported that alcohol misuse was present in the history of 56% of individuals who completed suicide [43].

Preuss *et al.* [87], in a large study involving 3190 individuals with alcohol dependence, demonstrated an association between suicide attempts and current situation of unemployment, separation or divorce and fewer years of education.

Reviewing the literature for the period 1991–2001, Cherpitel, Borges, and Wilcox [88] found a wide range of alcohol-positive cases for both completed suicide (10–69%) and suicide attempts (10–73%). Several case-control studies at the individual level have shown a high prevalence of alcohol abuse and dependence among suicide victims [89,90]. Kolves *et al.* in a psychological autopsy study reported that 68% of males and 29% of females who committed suicide met the criteria for alcohol abuse or dependence [89]. Strong support for a direct link between alcohol and suicide comes from aggregate-level data. Both longitudinal and cross-sectional aggregate-level studies usually report a significant and positive association between alcohol consumption and suicide [91–93]. Norstrom [94] reported that the estimated alcohol effect was stronger in Sweden (13% per liter) than in France (3% per liter). Ramstedt [57] studied the association for the period 1950–1995 in 14 European Union countries and found that an increase in drinking had a larger impact on suicide in northern Europe (8.6% per liter for men and 11.4% for women) than in mid-Europe and southern Europe (0.6% per men and 0.5% per women. If these data were to be confirmed, there is support for the hypothesis that the effect of alcohol on suicide rates is stronger in the northern European countries characterized by a low per capita consumption, with the bulk of consumption concentrated on a few occasions (binge-drinking pattern), or so-called “dry” drinking cultures, than in the southern European wine countries with a high average consumption that is more evenly distributed throughout the week, or so-called “wet” drinking cultures [95].

Razvodovsky [95] analyzed vodka sales in Russia. He reported that the coincident trends between the level of vodka sales and suicide rates in this period indicate that a restriction of vodka availability can be considered as an effective measure for suicide prevention in countries where rates of both vodka consumption and suicide are high. His time-series analysis suggests a positive relationship between the level of vodka sales per capita and suicide rates with no time lag and at first degree lags. As a matter of fact, the almost contemporaneous association between the two time-trends may support the point that binge-drinking of strong spirits is a risk factor for autodestructive behavior. It is important to point out that the size of the bivariate association between the level of vodka sales and suicide rates for men is substantially greater than for women. This means that alcohol-related suicide is mainly a male phenomenon, as was shown in previous studies [96,97]. Beverage preference and a harmful drinking pattern might be responsible for the gender difference in suicide rate as vodka continues to be the drink of choice for the majority of men in Russia, while women not only drink less often than men, but those who do drink consume vodka less frequently than men [98]. Follow-up studies suggest that alcoholics may be between 60 and 120 times more likely to complete suicide than those free from psychiatric illness [12]. Studies of samples of completed suicides indicate that alcoholics account for 20–40% of all suicides [99]. What is less clear is the role that alcohol plays in the events leading up to an act of suicide. It has been suggested that alcohol may influence an individual's decision to complete suicide, but few studies have investigated this possibility [100].

Post-mortem investigations have revealed that alcohol was in the blood of 45% of Swedish [101], 36–40% in Finnish [102,103], 35–48% of Estonian [104]; 28–29% of American [105,106] and 20% of Dutch [107] suicide victims.

An interaction has been found among alcohol use, impulsiveness and suicide risk [11,68]. The risk of suicide is higher in people with depression, particularly in cases of non-compliance or treatment-resistance, with alcoholism having the second highest risk. Among alcoholics, the lifetime risk of suicide is about 10–15%, and the majority of suicide attempts occurred in context of impulsiveness and alcohol abuse. Depression and/or alcoholism were comorbid in 85% of 100 cases of completed suicide [40], and this finding has been replicated more recently [43,108]. Cornelius *et al.* [109] stated that “patients who have alcohol dependence should be assessed for suicidal ideation whenever they exhibit significant level of other depressive symptoms, whenever they have a relapse of alcohol or drug use, and whenever they have experienced a recent interpersonal loss or a loss of housing or employment. Alcoholic patients with any depressive symptoms should be questioned carefully about symptoms that would define a major depressive disorder, because the presence of that comorbid disorder would be associated with a significantly increased risk for suicide”.

Bipolar disorder (BD) is strongly associated with high rates of suicide and suicide attempts. Alcoholism was associated with an increased rate of attempted suicide in 96 adult patients with BD-I, but not in 42 patients with BD-II, with or without a comorbid substance use disorder [110]. Earlier BD onset increased the likelihood of an association between alcohol use disorder and suicide attempts. The higher rate of attempted suicide associated with other drug use disorders appeared to be the result of higher impulsiveness, hostility and aggression. Co-occurrence of alcohol and drug use disorders increased the size of the association −97% of patients with BD-I and comorbid drug and alcohol use disorders had made a suicide attempt [110]. In another study, 99 of 239 patients with BD had a history of suicide attempts (41.4%). Of those positive for a history of suicide, only three were diagnosed with BD-II (3.03%) [111]. In this sample, borderline personality disorder and alcoholism were associated with past violent suicide attempts.

Wojnar *et al.* [112] investigated the correlates of impulsive and non-impulsive suicide attempts in 154 hospitalized patients with alcohol dependence. Lifetime suicide attempts were reported by 43% of the patients, 62% of whom scored high on impulsiveness. The only significant factor that distinguished patients making impulsive suicide attempts from patients making non-impulsive suicide attempts and with no suicide attempt was a higher level of behavioral impulsivity.

Simon *et al.* [113] found that individuals who made impulsive suicide attempts reported higher rates of aggressive behavior than those who made non-impulsive suicide attempts. They hypothesized that poor behavioral control, largely dependent on factors such as alcohol abuse, was an important indicator of risk for impulsive suicide attempts. Alcohol intake may result in a lack of behavioral inhibition and other aspects of impulsiveness, such as poor thinking and planning, as well as impaired attention.

However, despite higher rates of impulsive attempts and a higher level of lethality in patients with alcohol use disorders, the use of alcohol at the time of attempt did not differ significantly between impulsive and non-impulsive attempters [113–115].

Nevertheless, most of the literature presented is only correlational, and any causal conclusion is arbitrary and dangerous. In fact causality is a complex problem in the behavioral sciences [116]. Lipsey *et al.* [117], reviewing literature regarding alcoholism and violence, drew three general conclusions:

1. The research investigating the causal role of alcohol consumption in violent behavior is very unsatisfactory. As a result, the causal issue is still “cloudy and uncertain” (p. 277).
2. Despite the research fallacies, none of the relevant bodies of research yielded a consistently negative or null result on the causal question. Each provides substantial evidence of an alcohol-violence association that is consistent with a causal interpretation.
3. While the causal question may not be solved, it is apparent that there is a main effect of alcohol on violence. If alcohol has any causal effect on violence, it is true only in some people and/or some circumstances.

We think that these general conclusions of Lipsey *et al.* may be extended also to the question of the causal role of alcohol use and suicide. Alcohol use is neither a necessary nor sufficient condition for suicide, but may be regarded as a contributing factor. Furthermore, an inverse causal relationship (that suicidality may be a causal factor in alcohol use) has not been excluded. For example, Light *et al.* [118] investigated the link between alcohol use and suicidality with data from a longitudinal survey of junior and senior high school students from a suburban community in northern California. The results of the study indicate a possible causal relationship from alcohol problems to suicidality for adolescent males and a causal relationship from suicidality to alcohol problems for adolescent females. However, the results of Light and colleagues are limited by several factors. First of all, few, if any, of the young people in this study were problem drinkers or dependent on alcohol, and all of them were relatively early in their drinking careers. Second, suicide behavior was a rare event in this sample of adolescents. Third, the study was only observational, and the causal relationship may not be justified. The same drawbacks are evident in a more recent study [119]. In this study, Swahn *et al.* [119] examined the cross-sectional associations between preteen alcohol use initiation and subsequent suicide ideation and attempts for boys and girls in the 2005 national Youth Risk Behavior Survey, which includes a representative sample of over 13,000 high-school students in grades 9–12 in the United States. The authors concluded that preteen alcohol use initiation was significantly associated with suicidal ideation (adjusted OR = 1.89) and suicide attempts (adjusted OR = 2.71) relative to nondrinkers. However in the final multivariate model, an increased likelihood of suicide ideation and suicide attempts were also significantly associated with sadness, sexual assault, fighting, weapon carrying, and attending 9th grade (suicide attempts only). A negative association between suicide ideation and white students and students racially/ethnically classified as “other” was also noted. Similar results were obtained from analyses comparing those reporting teen alcohol use initiation to nondrinkers and preteen alcohol use initiation relative to teen initiation. Different conclusions were drawn by Chatterji *et al.* [35]. These authors used data from the 1991, 1993, 1995, 1997 and 1999 Youth Risk Behavior Survey samples, and a new estimation strategy proposed to address the issue of causal relationship between alcohol use and suicide. This study suggest that a causal relationship between binge drinking and suicide attempts is very unlikely, although a causal relationship between clinically defined alcohol use disorders and suicide attempts among female adolescents may occur.

### 3.4. Pathophysiological Mechanisms:a Neurobiological Link between Alcohol Misuse and Suicide

A direct relationship between alcoholism, suicidal behavior and specific changes in cerebral areas is not easily traceable. Recent studies of brain functioning in alcohol-dependent adults have produced varied results. However, it is well known that alcoholism is associated with dysfunctions in multiple neurotransmitter systems, and alcoholics are at significantly higher risk for suicide than individuals in the general population.

The main neurotransmitter systems involved in the action of alcohol are the GABAergic, the serotonergic and the glutamatergic systems but, secondarily, other bioaminergic transmissions may be involved, such as the dopaminergic and the noradrenergic systems [120,121]. The effects of ethanol, either acute or chronic, are due to the interplay of these neurotransmitters. These neurochemical actions need to be carried-out in specific brain nuclei for the multiple and fleeting alcohol-induced symptoms to occur. Combined brain imaging and neurochemical techniques may aid in assessing which molecules, which brain areas and which brain activities are affected. To see whether suicidal ideation, suicide attempts or having completed suicide might be associated with the same mechanism, the combined imaging-neurochemical techniques or post-mortem investigations should focus on the same target. At the neurochemical level, alcohol increases the activity of GABA, the brain’s principal inhibitory neurotransmitter [122], and other central brain mechanisms related to behavioral activation such as increased serotonin [123]. The well-known action of ethanol on GABAergic transmission is brought forth through various mechanisms and is region-specific [124,125], dose-related [126], and linked to both alcohol-induced behavioral inhibition and reward [127]. The activation of corticotropin-releasing factor type 1 (CRF1) receptors in the central amygdala appear to be crucial for the potentiation of GABAergic activity [128]. In this site, ethanol promotes the expression of new functional opioid receptors on both glutamatergic and GABAergic synapses which mediate ethanol-induced conditioned place preference [129] and inhibits acutely, but enhances chronically, non-NMDA pre- and post-synaptic glutamatergic activity possibly related to reward [130]. Another nucleus which is important in reward is the nucleus accumbens, which is rich in dopaminergic nerve endings and appears to constitute a final common target of most recreational drugs. In this nucleus, ethanol inhibits the presynaptic release of glutamate through opioid mechanisms and reduces post-synaptic glutamatergic transmission through inhibition of both NMDA-mediated and kainate-mediated currents [131].

Generally, it is believed that the sedative effects of ethanol are mediated by its combined action of both GABA transmission enhancement and glutamate transmission attenuation [132]. However, this is not always the rule. In the nucleus accumbens, core, metabotropic mechanisms enhance ethanol-mediated GABA activation [133], indicating complex interactions at that level. The nucleus accumbens is the terminal area where ethanol may interact with dopaminergic activity and reward, but an action on the origin of these neurones in the ventral tegmental area has also been described. This is mediated through the enhancement of local GABAergic transmission [134]. GABAergic enhancement is brought about by ethanol by stimulating sensitive neurones which bear GABA receptors with a alpha4/6/beta3delta channel structure, which are sensitive to the benzodiazepine analog Ro15-4513 [135].

Ethanol-induced NMDA inhibition in the cerebral cortex results in the reduction of noradrenaline and acetylcholine [136], and this might be related to the development of depression. This has been proposed as an explanation of the association between alcohol and depression, but may be also relevant to suicide. Glutamate in the cerebellum increases the levels of BDNF *via* NMDA, and this in turn reduces apoptosis. Ethanol decreases the effect of glutamate on BDNF [137] and may thus indirectly be related to the increased apoptosis and movement disorder found in chronic alcoholism. Interestingly, suicidal behavior has been found in a man with cerebellar agenesis [138]. Reduced serotonin function has been identified in suicides and possibly in serious suicide attempters (see [139] for a review) and alcohol dependent patients [140]. Serotonin depletion was also found in individuals displaying aggressive and impulsive behavior [139] and was a predictor of both early-ons*et al.*cohol use disorders [141] and suicide attempts among alcoholics [142,143]. Koob and LeMoal [144] suggested that the changes in hedonic tone that accompany substance use are central aspects of the addictive process, and the maintenance of substance use in the dependent person is driven by attempts to regulate the affective disturbance that results from substance use. Ethanol has been shown to potentiate acutely 5-HT3 receptor function and to modulate chronically 5-HT3-augmented mesolimbic dopaminergic function, but also to regulate alcohol drinking and its reinforcing properties at the ventral tegmental area level [145,146]. However, 5-HT3 receptors were not found to be altered postmortem in suicides [147]. An association between suicide attempts, impulsiveness, alcohol dependence and serotonin neurotransmission deficiency has been proposed [148–151], but the possibility that impulsive suicide attempts may reflect mood disturbance-related low serotonin activity in alcohol-dependent individuals is currently only speculative.

Neurobiological, including serotonergic mechanisms may play a role in the higher suicidality of depressed individuals with comorbid alcohol dependence compared to depressed subjects without comorbid alcohol dependence [152–155]. A study of prolactin responses to fenfluramine administration in patients with comorbid major depression and alcohol dependence, patients with major depression only and healthy controls found that controlling for gender, prolactin responses were lower in the comorbid group compared to the major depression only group or the health control group [156]. Controlling for gender and aggression, prolactin responses in the comorbid group remained significantly lower compared to the control group but the difference between the two patient groups disappeared which indicates that the difference in prolactin responses between the patient groups could be attributed to higher aggression scores in the comorbid group compared to the major depression only group. Another study found an anterior medial prefrontal cortical area where subjects with comorbid major depression and alcohol dependence had more severe hypofrontality than patients with major depression only [157]. This area encompassed the left medial frontal and left and right anterior cingulate gyri. This group difference disappeared after fenfluramine administration which suggests that serotonergic mechanisms play a role in the observed differences between the groups. Reduced serotonin input in the prefrontal cortex may underlie decreased behavioral inhibition in individuals with alcoholism and a greater probability of acting on suicidal feelings. A comparison of high- and low-lethality drug-free depressed suicide attempters with comorbid alcoholism showed that CSF 5-HIAA levels were lower in high-lethality attempters compared to low-lethality attempters which suggests that higher lethality of suicidal behavior in depressed patients with alcoholism is related to lower serotonergic activity [158]. Higher suicidality in depressed patients with alcohol dependence compared to depressed persons without comorbid alcohol dependence may also be related to the differences in dopaminergic regulation between the two groups. It has been observed that depressed subjects with a history of alcohol dependence had lower CSF HVA levels, compared with depressed subjects without a history of alcoholism [159].

Underwood *et al.* [160], using quantitative autoradiographic experiments in human postmortem brain tissue, found that binding to 5-HT1A receptors is lower in both alcoholic suicides and nonsuicides; so they suggested that this effect might be related to alcoholism and not to suicide. In nonalcoholic suicides, there is a localized increase in 5-HT1A binding in ventral prefrontal cortex, hypothesized to be a response to less serotonin input which had been hypothesized to be related to increased impulsivity and emotional dysregulation [161]. Recently, Oscar-Berman *et al.* [162] reported that alcoholics exhibit behaviors associated with prefrontal brain dysfunction in a study of 345 subjects in whom alcoholism and specific drinking variables (amount and duration of heavy drinking) significantly predicted frontal system and affective abnormalities.

Alcoholic suicides may fail to up-regulate ventral prefrontal 5-HT1A receptors in response to decreased serotonergic transmission increasing the risk of suicidal behavior. Binding to the serotonin transporter is low only in alcoholic suicides, suggesting an association with suicide. Evidence of impaired serotonergic innervation associated with alcoholism is also confirmed by less 5-HT1D terminal autoreceptor binding in alcoholics.

Furthermore, Storvick *et al.* [163] reported a decrease of the serotonin transporter density in the perigenual anterior cingulate cortex in the Cloninger type 1 alcoholics (prone to anxiety) using postmortem whole-hemisphere autoradiography. They also found that the 5-HT(1A) density was significantly decreased in the upper level of the perigenual anterior cingulate cortex.

Regarding the noradrenergic system, alcoholics had less alpha2 and beta1 adrenergic binding but more alpha1 adrenergic binding in the ventrolateral and orbital cortex [160]. Tapert *et al.* [164] found that alcohol-dependent women showed less differential response to working memory than controls in frontal and parietal regions, especially in the right hemisphere.

Regarding other receptors involved in the action of ethanol, genetic polymorphisms have been found in suicidal persons for both the CRF1 [165] and CRF2 receptors [166], but the latter is not apparently involved in the action of ethanol [127]. However, mRNA for CRF1, but not CRF2 receptors, were found to be reduced in the frontal cortex of suicides, along with mRNA for the alpha1, alpha3, alpha4, and delta receptor subunits of the GABAA-benzodiazepine receptor cortex [167]. It has to be mentioned, however, that CRF receptor numbers and affinity have been reported to be either reduced [168] or unchanged by different groups of investigators [169].

Altered glutamatergic receptors in the brains of people who died from suicide comprise reduced NMDA receptors [170] and increased caudate metabotropic receptors [171]. These findings are interesting in pointing to alcohol-suicide commonalities in neurochemical alterations but, unfortunately, these post-mortem findings in the brains of suicides are only partially matched by alterations found in brains of non-suicidal people with chronic alcoholism. Notably, GABAA receptors were reduced [172–174], but the subunit compositions only partly overlap with those found in suicides.

Regarding functional or structural neuroimaging studies, there is no overlap, with structural imaging in alcohol-abusing or -dependent people focusing on gross alterations such as ventricular enlargement and cortical thickness and research on suicidality consisting mainly of the detection of subtle abnormalities, such as white matter volume and hyperintensities [175,176]. Taken together, these results remain highly suggestive, but not conclusive, for a neurobiological link between alcohol misuse and suicidal behavior.

### 3.5. Proposed Psychological [or psychopathological] Mechanisms Explaining the Association between Suicide Behavior and Alcohol Use

A state of intoxication may trigger self-inflicted injuries, not only by increasing impulsivity, but also by promoting depressive thoughts and feelings of hopelessness, while simultaneously removing inhibiting barriers to hurting oneself [177]. Indirect mechanisms, including alcohol consumption as a form of self-medication for depression, or alcohol use as a marker for other high-risk behaviors, may also be relevant. Although we are far from understanding the relationships between alcohol use and suicidal behavior, a number of possible direct mechanisms for the association have been proposed.

Additionally, cognitive constriction (narrowed attention which reduces perceived potential solutions to a dichotomy—finding an immediate solution or committing suicide) is frequently observed prior to a suicide attempt [178]. Alcohol produces cognitive constriction through alcohol myopia [179], and this process has been confirmed by research showing that inhibition conflict (weighing pros and cons and identifying alternative solutions) mediates the relation between intoxication and social behavior [180].

Once a decision has been made to attempt suicide, alcohol use may serve several functions. Alcohol expectancies play an important role in determining alcohol use and behavior [181] and, consequently, it is reasonable to hypothesize that suicide-related alcohol expectancies relevant to gaining courage, numbing fears or anesthetizing the pain of dying may lead to the incorporation of alcohol use into a suicide plan. Alcohol may also serve as a “means to an end” as the suicide method itself [182–184].

There is a vulnerability to the neurotoxic effects of alcohol, and adolescent substance use has adverse consequences on brain development and executive functioning [185]. Some mood disorders may be alcohol induced (and remit after a period of abstinence) [186], and alcohol withdrawal is often associated with affective disturbance [187].

Sociological interpretations include the hypothesis that acute alcohol use leads to increased social deterioration and anomie [177], unemployment, debts, and social isolation [188–190]. Biological interpretations of the association include impaired physical and mental functioning [191] and interactions with other psychotropic drugs [192]. Disinhibition, in which alcohol acts to remove psychological and even physiological barriers to self-harm, has also been proposed as a relevant factor [193].

Hufford [194] summarized four psychological pathways for the proposed relationship between acute alcohol use and suicidal behaviour: (1) increasing psychological distress, including hopelessness, loneliness and depression; (2) enhancing or facilitating aggressive behaviour, including self-aggression; (3) changing an individual’s expectations and helping to propel suicidal ideation into action; and (4) constricting attention and inhibiting effective coping strategies that would facilitate avoiding suicidal behaviour. Rossow [195] has also reviewed a number of possible mechanisms in individual and aggregate level studies. However, these mechanisms are presented as *post hoc* interpretations of findings rather than as hypotheses tested in carefully designed studies, and so the evidence supporting these proposed pathways is limited.

## 4. Suicidal Behaviour and Alcohol Abuse in Special Populations

### 4.1. Suicide and Alcohol Abuse in Adolescents

A major public health problem is suicidal behaviour in children and adolescents. Suicide rates rise throughout the teenage years, especially in males. Suicide among adolescents constitutes 6% of all suicides and is the second or third cause of death in adolescents. According to data from the Youth Risk Behavior Surveillance study [196], 16.9% of high school students had seriously considered attempting suicide, 11.3% had made a suicide plan, and 8.4% had actually attempted suicide in 2005. These figures changed slightly in the 2007 report [197]; serious consideration of attempting suicide fell to 14.5%, making a suicide plan rose to 13%, and the actual suicide attempt rate fell to 6.9%. Whether these small changes are due to the enforcement of preventive programs is hard to say at present. The changes appear to follow a decreasing trend, in which considering suicide declined from 29% in 1991 to 14.5% in 2007 [197]. Interestingly, a similarly steadily decreasing pattern is shown for alcohol consumption in youths, although of lesser degree, going from 82% lifetime alcohol use in 1991 to 75% in 2007 [197]. Alcohol use in youths was associated with increased mortality and an increased death rate from vehicle accidents when driving while drunk [95], and this behaviour might constitute in some cases unconsciously motivated (or subintentioned) suicide attempts. According to Petersen [198] 20% of youths between 14 and 19 years of age have had thoughts about their own death, and 40% of youths who do not succeed in their first suicide attempt repeat the attempt. The rates of attempted suicide range from about five cases per 100,000 per year in 10–12 year-olds, 6 for 20–24 year-olds to 15 for those over the age of 65.

Singh *et al.* [24] reviewed autopsy and field reports for all paediatric suicide cases referred to the New Mexico Office of the Medical Investigator from 1979 to 2005. The age-adjusted suicide rate was 4.8 per 100,000 per year. Seventy-six percent of the suicides occurred in the victim’s home or yard. Shooting was the most common method overall (58%), followed by hanging (30%). In 26% of the cases, alcohol or other drugs were detected post-mortem. The results of toxicology testing were more often positive in decedents over the age of 15, and rare in suicides younger than 15. Males were 2.7 times more likely to have an alcohol use disorder than were females. Those who shot themselves were 2.4 times more likely to have an alcohol use disorder than those who hanged themselves or used other methods. Boys were more often intoxicated at the time of suicide than were girls. However, although alcohol or illicit drug use is frequently cited as a risk factor for suicide, the authors reported a low prevalence of intoxication, again suggesting that suicide is not simply (or not often) the result of an impulse. Therefore, the use of suicide as a way of solving a chronic problem rather than an impulsive response to stress means that prevention programs based on impulse control, such as crisis intervention, will be less effective in this population. However, impulse reduction may reduce self-damaging acts and, *de facto*, contribute to a reduction in self-inflicted mortality, be it suicidal in nature or not.

Although the age-adjusted suicide rate was higher in New Mexico than nationally, the trends in the overall population remained similar. In a recent case-control study on the utilization of health care services prior to suicide (across different time periods) among children and adolescents aged 11 to 18 years in the Province of Quebec, Canada, Renaud and colleagues [199] investigated the utilization of services (*i.e.*, contact with general practitioners, mental health professionals, psychiatrists and/or youth protection groups) at different time periods in 55 child and adolescent suicides and 54 matched community controls using proxy-based interviews and questionnaires. More than 90% of the child and adolescent suicides suffered from a mental disorder, and a significant proportion of them were left without appropriate healthcare support (including psychiatric consultation) in the period preceding their suicide. Furthermore, 20% of the suicides and no control subjects had made prior suicide attempts. More specifically, over two-thirds of the suicides had no treatment contact within the month prior to the suicidal action, while only 12.7% of them were in contact with psychiatric services during that same period. Females seemed to have more psychiatric and mental health service contacts in the most recent month, and those adolescents with depressive and anxious disorders had received more psychiatric and general mental health services in the past year. Past-month hospitalization was more often associated with alcohol abuse and psychosis. The authors highlighted the need for an overall increase in the rates of healthcare services delivered to young people at risk for suicide, as well as better training of health professionals in detecting and treating adolescent psychopathology. In this population of adolescents referred to healthcare services, at least a quarter of the suicides abused drugs and/or alcohol.

More worrying data comes from research reported by the CDC (Centers for Disease Control and Prevention) [200] indicating that 25.6% of high school students had drunk their first alcoholic drink by age 13, and 25.5% admitted having drunk five or more alcoholic drinks in a row within the last two weeks. Translated, this mean that one out of four youths regularly engages in binges and that about the same proportion has started taking alcohol early in their life when their brain is still maturating. Among people with depression, those who consumed substances or alcohol have a higher probability of attempting suicide as compared with depressed individuals who did not [201].

Many factors associated with suicide in adults are also present in younger people. Family transmission of suicide risk is important, especially when suicidal behavior has occurred on the maternal side [202]. Most young people who die by suicide have psychiatric disorders. Affective disorders, substance-related disorders and disruptive behavior disorders are the most frequent [203]. Other important contributing factors include previous suicide attempts, family disruption and discord, recent losses, physical and sexual abuse, homelessness, and a homosexual or bisexual gender orientation [203,204]. Media influences are important in young people [205], and some suicides occur in clusters, indicating a role for the “Werther effect” in adolescent suicide [206].

In light of the above evidence, it is difficult to attribute a role for alcohol in adolescent suicide. Patterns of alcohol consumption by youngsters in Western countries are changing, and singling-out the role of alcohol in suicide becomes an increasingly harder task, since alcohol use is increasing, as far as absolute numbers are concerned, while suicide changes very little in numerical terms. However, drinking alcohol has been used in human societies in ritualistic contexts and has a symbolic value, and it has maintained this role even when the formal framework has changed. Its anxiolytic properties help people in personal and social contexts in which they are confronted with difficulties. Alcohol becomes a way of facilitating communication with others and adapting to the environment. Suicide is also both a social and a personal act and is related to conditions that render life difficult. It is possible that when one decides to commit suicide, he/she may select one of the options available to make the act more socially and personally acceptable, and one of these may be alcohol. In doing so, the person communicates to others and adapts to his/her environment. The results of research do not support the hypothesis that, when a youth gets drunk, this in itself leads to that youth deciding to commit suicide.

### 4.2. Suicidal Behavior and Alcohol Abuse in Elderly People

In almost all industrialized countries, the highest suicide rate is found among men aged 75 years and older [207]. Whereas suicidal behavior in youngsters is often impulsive and communicative, in older people it is often long-planned and involves highly lethal methods. Its lethality increases also as a result of the structural frailty and loneliness that are often present in the elderly. Alcohol misuse is an important risk factor for suicide in elderly people [208]. Psychiatric disorders, especially depression, are common in suicides in Western [208], as well as in Eastern countries [209]. Depression on the other hand, is frequently comorbid with alcohol abuse/dependence in the aged [210–212].

In later life in both sexes, major depression is the most common diagnosis both in those who attempt suicide and those who complete suicide. In contrast to other age groups, comorbidity with substance abuse and personality disorders is less frequent [207]. Cognitive rigidity and obsessional traits seem to influence the risk of suicide in the elderly [213,214], probably because these traits undermine the ability of the elderly to cope with the challenges of ageing, which often calls for substantial adaptations. Physical illness [215], bereavement and loss of independence [216] are also important factors. Physical illnesses play an important role in the suicidal behavior of the elderly. Depression and physical illness are frequently comorbid. In many cases, the physical illness itself, and medications adopted to treat it, may cause depressive symptoms. About 2–4% of terminally-ill elderly patients commit suicide. Complicated or traumatic grief, anxiety, unremitting hopelessness after recovery from a depressive episode, and a history of previous suicide attempts are risk factors for attempted and completed suicide. Overt suicidal behavior and indirect self-destructive behaviors, which often lead to premature death, are common, especially in residents of nursing homes, where more immediate means to commit suicide are restricted.

The alcohol-suicide-psychiatric disorder/psychological distress link in the elderly may differ from the link in those of other ages. In the elderly, the highest incidence of suicide ideation is found among the lower age ranges, and at-risk alcohol use appears to be less accompanied by such ideation as compared to the psychiatric disorders such as depressive and anxiety disorders [217] whereas, in young people, suicidal ideation is associated with both depression and alcohol abuse [218].

### 4.3. Suicidal Behavior and Alcohol Abuse in Affective Bonds and Social Relationships

Alcohol use disorder has an enormous impact on relationships, generating ambivalence and anger. These effects are even stronger in the face of suicidal behavior. According to Wasserman [219], many suicidal persons with alcohol dependence have borderline personality disorder. They have contradictory affective reactions and are often confused as to whether others love or hate them and whether they love or hate others. They have difficulty in distinguishing between the good and evil impulses in themselves and other people. Copello *et al.* [220], after reviewing the literature, concluded that people with drug and alcohol use disorders often behave in ways destructive to family life and relatives.

Velleman and Templeton [221] noted that family members (children, partners, siblings, parents and other close relatives) are often affected by this. Wolk-Wasserman [222] found that suicidal, substance-abusing patients commonly break-off relations with a significant other before committing suicide and increase their suicidal communications during the break-off period. Marshal [223] tested two competing hypotheses relating alcohol use disorder and marital functioning: (1) that alcoholism disrupts and ends marriage, and (2) that alcohol use disorder is secondary to marital dysfunction and tends to stabilize marriage and perhaps prevent its dissolution. His results found no support for the latter hypothesis, supporting rather that alcohol use is maladaptive and is associated with dissatisfaction, negative marital interaction patterns and higher levels of marital violence.

Wolk-Wasserman [222] found that the suicidal hints or threats were usually not taken seriously by the partners of those with alcohol dependence, even when suicide had been attempted previously. Parents of substance-abusing suicide attempters fear that their children will commit suicide, which makes them desperate [222]. They often accused their partners of causing their children’s troubles and reproached social service and psychiatric authorities for failing to look after them properly.

Velleman and Templeton [221] described the impact of parental substance use disorder on adolescents and young adults. They concluded that the offspring of persons with alcohol dependence are particularly likely to report being detached, switching-off, avoiding the drinking parent and blaming themselves. Offspring described a variety of ways of escaping childhood adversity, such as leaving home significantly earlier than others.

The spouses of suicides who misused alcohol were significantly more likely to react with anger than the spouses of those who did not. The children of parents with alcohol use disorder who completed suicide were less likely to feel guilty or abandoned than the children of non-alcohol-related suicides. Alcohol use disorder before suicide changes the affective responses in the spouses and the children who are left behind. Survivor reactions to suicide are strongly influenced by the nature of the relationship between survivors and the suicide. Bereavement counsellors should be alert for complex grief and mourning responses among this group of suicide survivors.

Emotional reactions in survivors differ, with spouses and parents significantly more affected than adult children [224]. Parents showed more sorrow, depression, feeling of powerlessness and guilt, while spouses felt more abandoned and angry [224]. Their anger is directed to the lost person significantly more than that of spouses whose suicidal partner had no alcohol problems [225]. Alcoholism in any close relationship causes tension and conflicts and complicates bereavement.

Further research in needed to address the impact of the quality of the relationship, emotional attachment, age (of the survivor and the suicide) and other factors on bereavement.

## 5. How to Deal with the Problem: Preventive Measures and Future Perspectives

Prevention is the “logical outcome” of suicide research [28]. Suicide prevention is primary with respect to alcohol use, but must take into account the alcohol abuse especially in cases where the alcohol use facilitates suicide behavior.

### 5.1. Prevention of Suicide by Focusing on the Alcohol Abuse Component

Several countries have established national suicide prevention strategies which include specific targets for the reduction of suicide. Suicide prevention strategies are targeted at both high-risk groups (selective or indicated) and general population (universal interventions) [226].

Although groups at risk can be identified, the prediction of suicide in individuals is difficult because individual risk factors account for only a small proportion of the variance in risk and lack sufficient specificity, resulting in high rates of false positives [227]. The management of people at risk of suicide is challenging because of the many causes and limited evidence base.

Universal preventive interventions are directed to the entire population, selective interventions target people at greater risk for suicidal behavior, and indicated preventions are targeted at individuals who have already exhibited self-destructive behavior. People with psychiatric disorders, alcohol and/or drug abuse, newly diagnosed severe physical illness, past suicide attempts, homelessness, institutionalization, and other types of social exclusion are the object of selective interventions.

People with depression should be screened for suicide risk by being asked specifically about suicidal thoughts and plans. If suicidal ideation is present or if suicidal intentions are suspected, risk factors for suicide should be assessed. If suicide risk is present, further assessment should address the imminence of suicidal behavior. Intention to die (explicitly expressed or inferred from behavior), cogent plans, and high levels of hopelessness might indicate imminent risk. This risk is likely to be heightened by alcohol misuse and easy access to methods by which to carry out a suicidal act. In cases of high or imminent suicide risk, immediate action is needed, including hospitalization, removal of potential methods of suicide, and initiation of vigorous treatment of the associated psychiatric disorders.

The diagnosis of depression is crucial for suicide prevention because treatment of unipolar depression is different from that of bipolar depression, the latter increasing the likelihood of suicide if treated only with antidepressant drugs [228–232]. Incomplete symptomatology, impulsive actions, periodic alcohol abuse, compulsive buying behaviors, acute delusional episodes, medicolegal actions and comorbidities can hide or modify bipolar symptomatology. Bipolarity should be systematically screened for in cases of substance abuse (present in 40–60% of bipolar disorder patients), particularly in cases of alcohol abuse [233]. Regulatory agencies have issued warnings that the use of selective serotonin-reuptake inhibitors poses a small but significantly increased risk of suicidal ideation or nonfatal suicide attempts for children and adolescents [232,234]. Guidelines recommend that antidepressants should be given only to moderate or severely depressed adolescents and only combined with psychotherapy [235].

The evidence about the consequences of antidepressant treatments in subjects with comorbid alcohol dependence and mood disorders was unclear and not well documented. Recently, Ukai *et al.* [236] reported that treatment with antidepressants reduced the alcohol-induced suppression of neurogenesis, and Umene-Nakano *et al.* [237] found that depressive patients suffering from both depression and alcohol dependence responded to 8 weeks of treatment with antidepressant drugs and had significantly increased serum BDNF levels. However, many factors may influence the response to antidepressant treatment, such as the age at onset of the first major depressive episode which was significantly correlated with the treatment response to escitalopram in patients with major depression and comorbid alcohol dependence [238], and the differential treatment effects based on alcohol dependence comorbidity have been understudied [239]. Cornelius *et al.* [240] found that the long-term clinical course for major depression in the comorbid adolescent population is surprisingly poor also including a higher mortality from suicide and higher treatment costs [241]. The poor response to antidepressant treatment was found to be an independent risk factor for suicide attempts in 1,863 persons included in the WHO/ISBRA study; 292 of these patients had both a history of depressive symptoms and alcohol dependence or abuse [242].

So, the poor antidepressant treatment response in subjects with co-morbid alcohol dependence and depression, or only with alcoholism, may have important negative effects also, such as increasing suicidality.

Excluding substance-induced psychotic disorders, the lifetime rate of substance use disorders in people with psychotic disorders is 62.5%. Alcohol, amphetamine and cannabis are the dominant substances. Alcoholism may cause acute paranoid-hallucinatory psychosis and, although prognosis is good, 10–20% of patients with alcohol psychosis will develop a chronic schizophrenia-like syndrome [243,244]. Strategies for patients with psychoses must take into account the fact that alcohol dependence and psychosis, which alone are risk factors for medical problems, multiply the risk when comorbid [245].

In dealing with people with alcohol and/or substance abuse, male gender, older age, serious violent or other past suicide attempts, somatic and mental disorders, suicide planning or high suicidal intent, and receiving psychiatric treatment must be considered, as they are all positive predictors of a new suicide attempt [246]. People in whom any one of these risk factors is present should be monitored carefully. Since most suicides associated with psychiatric hospitalization occur shortly after admission (mostly through hanging) or after discharge, safer environments, intensive clinical care and ongoing care beyond the point of clinical recovery are important in order to reduce the risk of suicide [247]. The relationship between alcohol/drug use and the degree of suicidality in acute psychiatric patients is strong [248]. Among these patients, the most severely suicidal have a high rate of alcohol abuse/dependence (56%). Furthermore, in 40% of these patients, substance/alcohol use preceded and/or caused the hospitalization. Repeated self-harm or attempted suicide are positively associated with a higher suicide risk [249]. Therefore, people with such antecedents should be targeted in prevention programs. In fact, improved treatment may be a key factor in suicide prevention in patients during and shortly after hospitalization for affective disorders [250].

Reducing alcohol consumption, thereby rendering the person less abusing and less dependent, may focus on socially reinforcing the sober condition rather than blaming alcohol intake. Increasing the person’s social acceptance is one of the means to reduce suicide thinking. Other tactics should focus on self-esteem. In fact, people with alcohol abuse often are afflicted with self-blame and may feel rewarded or vindicated when the self-fulfilling prophecy of being rejected is realized. Fostering and strengthening positive values may indirectly reduce suicide risk by rendering life more pleasurable.

There is a high alcohol abuse disorder comorbidity rate in schizophrenia. Uzun *et al.* [251], investigating the possible association between socio-demographic and clinical variables and suicidal behavior in a sample of 300 patients diagnosed as having schizophrenia in Turkey, found that the suicide attempters tend to be younger age at onset of the disorder have a longer duration of psychosis and more hospitalizations, and are more likely to have lifetime major depressive episodes and a significantly higher rate of alcohol abuse or dependence than patients without a lifetime history of suicide attempts. McGirr *et al.* [252] reported that, compared to other suicides, schizophrenic and schizoaffective suicides showed comparably elevated levels of impulsive aggressive traits. Evren and Evren [253] found that, among schizophrenic patients, young male patients who have antisocial personality properties and depressive symptoms should be considered at higher risk for suicide.

Using the psychological autopsy method, Heilä *et al.* [254] investigated schizophrenic subjects in whom active illness and depressive symptoms were highly prevalent immediately before suicide and a history of suicide attempts was common. Women were more likely to have committed suicide during an acute exacerbation of the illness. Alcoholism was most common among middle-aged men (45%), whereas middle-aged women had a high rate of depressive symptoms (88%). They noted that younger male subjects most often used violent suicide methods.

Bartels *et al.* [255] reported that alcohol use was also correlated with depression and suicidal behavior, and depression alone accounted for over 80% of the explained variance in suicidal behavior. Alcohol use alone and the correlation between depression and alcohol use accounted for only small amounts of variance.

Thus, alcohol abuse may affect the risk for suicide in schizophrenia, but several factors may be critically involved in this association.

There is a high comorbidity rate in major depression from alcohol abuse disorder. Sullivan *et al.* [256] found that alcohol problems are more common in those with major depression than in the general population and are associated with adverse clinical and health care utilization outcomes. In addition, antidepressants may be effective in the presence of alcohol dependence. Dumais *et al.* [257] found that current (six-month prevalence) alcohol abuse/dependence, current drug abuse/dependence and cluster B personality disorders increased the risk of suicide in individuals with major depression. Also, higher levels of impulsivity and aggression were often associated with suicide in major depression.

Aharonovich *et al.* [258] found that all subtypes of depression increased the risk for making a suicide attempt in patients with substance dependence abuse. Major depression occurring before the patient became substance dependent predicted the severity of suicidal intent, while major depression during abstinence predicted the number of attempts.

Thus, the relationship between alcohol abuse and depression in determining suicidality is complex and multifaced, and there are many factors which may impact on suicidality in depressed patients.

### 5.2. Implications for Prevention

The high rate of suicide among adolescents and young adults is a challenge for prevention. The CDC’s National Center for Injury Prevention and Control [259] published guidelines for the development of intervention strategies for communities interested in adolescent and youth suicide prevention programs. The strategies focus on identifying youths at risk so as to direct them to healthcare centres, defining the risk factors, and providing support to manage stressful life events. The guidelines recommend making sure that suicide prevention programs are strongly linked with the mental health resources in the community. A good prevention program should adopt a broad spectrum approach since suicide cannot be explained with linear cause-and-effect logic, but rather as a complex and multidimensional phenomenon. The guidelines also recommend incorporating promising, but underused, strategies into current programs where possible, expanding suicide prevention efforts for adolescents and young adults, introducing screening programs, and evaluating the prevention programs.

In clinical contexts, patients often avoid mentioning their suicidal ideation, but they are more willing to discuss it if the doctor asks specific questions about their suicidal intentions. Therefore, giving information and training to general practitioners and nurses may have an enormous impact on how the patients at risk are evaluated and managed. This may be useful also for teachers, parents, relatives and all those who come into contact on a regular basis with at-risk individuals.

Summarizing, one of the most effective strategies for suicide prevention is to teach people how to recognize the cues for imminent suicidal behavior and to encourage youths at risk to seek help. Antisocial traits and substance abuse (including alcohol abuse) are strongly connected to suicide. It is important that psychiatric disorders in youths are immediately diagnosed and treated.

### 5.3. Implications for Intervention: A Focus on the Individual Context

Any kind of intervention should consider the individual context in which alcoholism, substance abuse and suicidal behavior appear. Attempted suicide is a markedly deviant behavior, and deviant behaviors often lead to peer rejection, which in turn can lead to a tendency to associate with other deviant peers [260] and also to use and abuse substances, including alcohol [261]. A suicide attempt may be followed by profound modifications of the attempter’s environment one to two years after the attempt. For instance, 58% of adolescents who attempted suicide no longer live in their parent’s home [262], while 74% experience reduced school performance after the attempt [263]. Spirito *et al.* [264] reported that 23% of adolescents who attempted suicide were subsequently involved in violent acts within three months after hospitalization. Additionally, college students who attempt suicide are often not allowed to continue their studies due to fears of litigation [265,266]. Finally, adolescent psychiatric hospitalization is perceived as stigmatizing by family members [267], and this perceived devaluation–discrimination can lead to decreased self-esteem among people with mental illness [268]. In such cases an unsuccessful suicide attempt may lead to problematic drinking in order to self-medicate [269], as well as negative feelings and poor social outcome. Furthermore, Dalton *et al.* [270] in their sample of bipolar patients found that that is drug abuse and dependence (but not alcohol abuse and dependence) was associated with increased risk of lifetime suicide attempts. Among the selected clinical predictors of suicide attempts, rapid-cycling was the only other predictor of suicide attempts. One factor underlying the association between substance use disorder and suicide attempts may be severity of illness: subjects with a more severe form of bipolar disorder may be more likely to both attempt suicide and use substances to self-medicate. This theory is partially supported by the finding in the present study that subjects with substance use disorder had an earlier age of onset of BD. Not only is age of onset a recognized indicator of severity of illness, the connection between both alcoholism and substance abuse and earlier age of onset of BD has been confirmed by other studies. The severity of illness theory also offers a possible explanation for the lack of association between alchol use disorder and suicide attempts. Given that drugs, but not alcohol, are illicit and therefore more difficult to obtain it is reasonable to speculate that patients who abuse drugs have more severe overall mental health problems, than patients who abuse only alcohol. Moreover, Personality traits, in particular impulsivity, may also explain the association between suicide attempts and substance related disorders. Impulsivity is a prominent element of manic episodes, is elevated in substance-dependent individuals and is an established risk factor in both suicide attempts and completed suicide in affective disorders. It may be hypothesized that suicide attempts and co-morbid SUD occur in bipolar patients who have higher levels of trait impulsivity.

Intervention should help people find a motivation to stop drinking, identify the circumstances that motivate them to drink, identify the factors that engender this conduct, and evaluate the possible risk of suicide. Psychotherapy can help individuals learn new methods of coping with stressors and develop social relationship in the community. Particularly for adolescents, family therapy may play a crucial role both in the resolution of the problems and in the recovery of the patient.

The need for primary prevention programs seems clear and, despite focusing on late-stage prevention such as crisis intervention, such programs should address the underlying risk factors for suicide: mental health, social competence, conflict resolution, problem-solving, and family and community support. Pessimism about the future may be related to the individual’s social environment, life experiences and exposure to adverse factors, such as poverty, unemployment, prejudice and discrimination.

### 5.4. Future Perspectives

Because suicide is a complex problem, no single approach is likely to contribute to a significant, substantial decline in suicide rates. Clinical studies of suicide prevention are hindered by methodological and ethical problems, especially since many people at risk do not have contact with clinical services. Knowledge about who is at risk of suicide is crucial, and a number of interventions show promising effects. Future research must focus on the development of suicide-prevention based on specific assessment and treatment protocols.

## 6. Concluding Remarks

**Figure 1.**
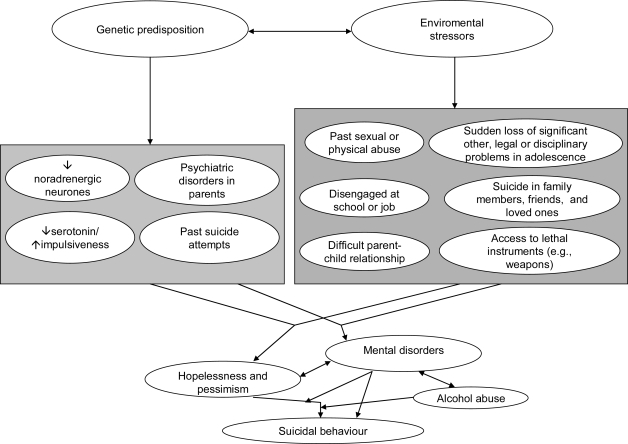
The complex relationship between risk factors and suicide.

Suicide is the result of complex interactions between biological, psychological, social and environmental factors (Figure 1), and all of these conditions impact on one another. Environmental stressors act on a genetically-determined and environmentally-modulated physical structure that in turn impacts psychological well-being and may cause a psychiatric illness that affects the person’s inner world and paves the way for suicide. Alcohol abuse is a means of easing one’s psychological stress but, at the same time, impacts on all other factors, rendering suicide more likely.

Depression is frequently a precursor of alcohol abuse, but alcoholism may also trigger or exacerbate depression. Suicidal behavior usually occurs early in the course of mood disorders, but only in the final phase of alcohol abuse when social marginalization and poverty, the somatic complications of alcoholism and the breakdown of important social bonds have taken over.

Attitudes toward and drinking and help-seeking behavior are culturally determined, but genetic factors play an important role in the predisposition to both suicidal behavior [271] and alcohol abuse [272,273].

Figure 1 indicates the impact of alcohol abuse and misuse on suicide risk and the importance of the detection and treatment of alcohol use disorders for suicide prevention. Therefore, suicide prevention should focus on the diagnosis and treatment of alcoholism [63] and other substance-related disorders. In view of the strong link between alcoholism and suicide, there is a clear need to provide public health education regarding sensible drinking. The well-established heritability of alcohol consumption and the interaction of genes with social and environmental factors [274] should also be taken into account when dealing with alcohol use as related to suicidal behavior. Failure to identify specific alcohol-related disorders can delay the initiation of readily available therapies and increase the morbidity and mortality of patients.

Further research is needed to examine specific subgroups at higher risk of suicide and to compare attempted suicides with completed suicides, to develop alternative risk-profiles and to devise intervention strategies that are robust enough to account for social and cultural differences. Suicide is a major public health problem and must be given high priority with regard to prevention and research. The cultural and biological underpinnings of alcohol use may have a preeminent place in this effort. Alcohol prevention programs may positively impact public mental health and help reduce suicide risk indirectly.
